# Capillary flow of liquids in open microchannels: overview and recent advances

**DOI:** 10.1038/s41526-021-00180-6

**Published:** 2021-12-09

**Authors:** Panayiotis Kolliopoulos, Satish Kumar

**Affiliations:** Department of Chemical Engineering and Materials Science, University of Minnesota, Minneapolis, MN 55455 USA

**Keywords:** Mechanical engineering, Fluid dynamics, Chemical engineering

## Abstract

Capillary flow is the spontaneous wicking of liquids in narrow spaces without the assistance of external forces. Examples of capillary flow can be found in numerous applications ranging from controlling and transporting fuel in spacecrafts to printed electronics manufacturing. Open rectangular microchannels often appear in these applications, with the lack of a top resulting in a complex free-surface morphology and evaporation. Here, we present a brief overview of this topic and discuss some recent advances.

## Introduction

Capillary flow is the spontaneous wicking of liquids in narrow spaces without the assistance of external forces. This phenomenon is common in everyday life and industrial applications. Examples of capillary flow can be found in diverse fields, ranging from physiology, where capillary flow is essential for the drainage of tear fluid from the eye, to flexible printed electronics manufacturing^1–3^, where capillary flow is used to create electronic circuits that can be used in sensors for brain–machine interfacing. Additional applications include lab-on-a-chip devices^4,5^, heat pipes^6^, propellant management devices in spacecrafts^7^, paper-based microfluidics^8^, fuel cells^9^, porous-media flows^10,11^, and storage and handling of fluids and waste in low-gravity environments^12,13^. Understanding the mechanism of capillary flow and finding ways to control it has been a subject of investigation since the early twentieth century.

The purpose of this Perspective article is to provide a brief overview of and discuss some recent advances in our fundamental understanding of capillary flow for an important special case: rectangular microchannels without a top. Such open microchannels appear in a variety of applications and involve more complex flow behavior than their closed counterparts. In addition, the liquid in the microchannel may evaporate, leading to further challenges in understanding flow behavior. Notably, the channels involved have widths and depths of tens to hundreds of microns so that surface tension, i.e., capillary forces play a key role in driving flow. The importance of capillary flow and evaporation in open microchannels along with recent insights obtained through an interplay between theory and experiment^14,15^ make this Perspective article particularly timely, and we hope it will inspire others to advance both the fundamentals and applications related to this topic. We first discuss capillary-flow dynamics in the absence of liquid evaporation, and then consider the influence of evaporation.

## Capillary-flow dynamics

Early studies by Lucas^16^ and Washburn^17^ focused on developing theoretical models to understand the physical mechanisms driving spontaneous capillary flow in cylindrical tubes. Their models described the meniscus position ${\hat{z}}_{\rm{M}}$ as a function of time $\hat{t}$ for flow of Newtonian liquids. Lucas^16^ assumed the flow is driven by the capillary pressure gradient caused by the circular-arc meniscus front, while Washburn^17^ also included hydrostatic pressure gradients and an imposed pressure difference between the two ends of the cylindrical tube. For a horizontal cylindrical tube open at both ends, an analytical solution ${\hat{z}}_{\rm{M}}=\sqrt{\hat{k}\hat{t}}$ is obtained, commonly referred to as the Lucas–Washburn relation, where $\hat{k}$ is the mobility parameter and depends on the cylinder radius, liquid viscosity $\hat{\mu }$, surface tension $\hat{\sigma }$, and contact angle *θ*_0_. The mobility parameter can be thought of as a diffusion coefficient driving the growth of the liquid interface.

Numerous investigators extended the theoretical work of Lucas^16^ and Washburn^17^ by including inertial^18–20^, gravitational^21^, dynamic contact angle^22–24^, surface roughness^24,25^, wettability gradient^26^, electrowetting^27^, and non-Newtonian rheology^28,29^ effects. In addition, these theoretical models have been extensively compared to experiments^18,19,23,24,30–34^. Prior studies have identified two characteristic regimes for capillary flow in horizontal capillaries, where gravitational forces play a negligible role in driving flow. At early stages the flow is dominated by inertial effects, resulting in ${\hat{z}}_{\rm{M}} \sim \hat{t}$. At later stages, inertial effects are negligible, and viscous effects dominate, resulting in ${\hat{z}}_{\rm{M}} \sim {\hat{t}}^{1/2}$. Both scalings have been confirmed experimentally.

For vertical or inclined capillaries, gravitational forces can have a significant influence on the flow. As a result, at later times a third characteristic regime is observed where gravitational effects dominate, leading to an asymptotic plateau in ${\hat{z}}_{\rm{M}}$. In general, the relative importance of gravitational forces to surface-tension forces is given by the Bond number, ${\rm{Bo}}=\hat{\rho }\hat{g}{\hat{D}}^{2}/\hat{\sigma }$, where $\hat{\rho }$ is the liquid density, $\hat{g}$ is the magnitude of the gravitational acceleration, and $\hat{D}$ is a characteristic length scale. The discussion in this article is primarily focused on the case of vanishing Bo, where gravitational effects are negligible.

**Fig. 1:**
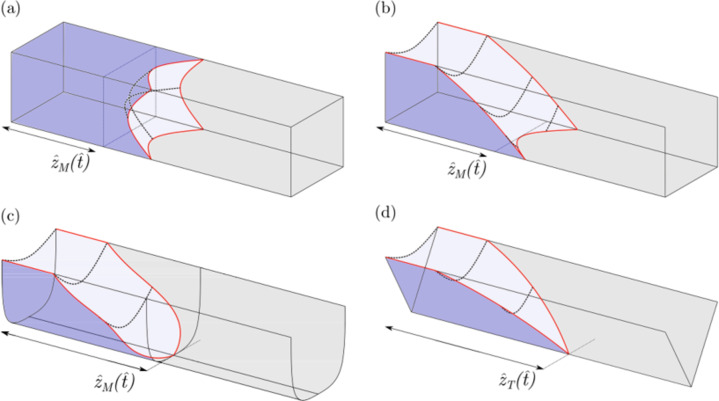
Microchannel shapes. Schematic of flow in (**a**) closed rectangular, (**b**) open rectangular, (**c**) open U-shaped, and (**d**) open V-shaped microchannels.

Microchannels can broadly be classified as closed (Fig. 1a) or open (Fig. 1b). A closed microchannel is defined as one where all walls are solid, whereas an open microchannel lacks a top. Due to breakthroughs in lithographic fabrication techniques, open microchannels with various cross-sectional geometries (Fig. 1b–d) can be fabricated easily and inexpensively, including rectangular^14,15,35–37^, trapezoidal^38^, U-shaped^35^, and V-shaped^39–43^ cross-sections. The lack of a top provides access to the inside of the channel and has been exploited in applications such as capillary micromoulding and microfluidics. In addition, the risk of clogging in open microchannels is reduced compared to their closed counterparts^5^.

Motivated by the advantages of open microchannels, investigators have examined capillary flow in these geometries using theory and experiment. Many studies modified the Lucas–Washburn relation, which was initially developed for cylindrical tubes, to model arbitrary cross-sectional geometries^24,44^. In these studies, while the scaling of ${\hat{z}}_{\rm{M}} \sim {\hat{t}}^{1/2}$ still holds, the expression for the mobility parameter $\hat{k}$ depends on the cross-sectional geometry. However, predictions from these modified Lucas–Washburn models (MLWs) for open channels have resulted in a varying agreement with experiments^14,15,24,35,36,38^. This is because the mechanism for capillary flow in open channels is more complex than that for closed channels. While for closed channels the force driving the flow is due to the pressure gradient caused by the circular-arc meniscus front (Fig. 1a), for open channels the additional free surface due to the lack of a top also contributes to driving the flow (Fig. 1b–d)^15^.

The additional contribution of the free-surface curvature to capillary flow has been theoretically and experimentally investigated primarily for V-shaped channels^39–42,45,46^. Spontaneous flow in V-shaped channels is observed if the liquid contact angle satisfies *θ*_0_ < *π*/2 − *β*, as described by Concus and Finn^47^, where *β* is the half-angle of the interior corner. The flow leads to the formation of filaments or fingers, where in horizontally oriented channels or in low-gravity environments the finger tip ${\hat{z}}_{\rm{T}}$ scales as ${\hat{z}}_{\rm{T}} \sim {\hat{t}}^{1/2}$^39–42,45,46,48^ and in vertically oriented channels in the presence of gravity it scales as ${\hat{z}}_{\rm{T}} \sim {\hat{t}}^{1/3}$^49,50^.

The most widely used open-channel cross-sectional geometry is rectangular because it is relatively simple to fabricate^4^. However, only a few previous theoretical studies have considered the additional contribution of the free-surface curvature to capillary flow in open rectangular microchannels. These studies primarily focused on the capillary flow of perfectly wetting liquids (liquid equilibrium contact angle *θ*_0_ = 0) in microchannels with large aspect ratios $\lambda =\hat{H}/\hat{W}$ (height/width)^51,52^ or reported three-dimensional simulations using the volume-of-fluid method to study the effects of gravity on the capillary rise in vertically oriented open rectangular channels^53^.

In open rectangular channels, the free-surface morphology is more complex than in U-shaped and V-shaped channels (Fig. 1). From the channel inlet to the meniscus front ${\hat{z}}_{\rm{M}}$ the upper meniscus spans the entire channel width. At the meniscus front, the flow splits into the channel corners provided the liquid equilibrium contact angle *θ*_0_ < *π*/4^47^. This splitting of flow leads to filaments or fingers extending ahead of the meniscus and influencing its propagation. Such a transition is not observed in U- and V-shaped channels. Note that although the meniscus and fingers are coupled to each other and part of a single free surface, it can be convenient to characterize their behavior separately. For example, Weislogel^48^ modeled the complex free-surface morphology in closed rectangular channels by coupling the advancing meniscus motion using a Lucas–Washburn-type model with the filament propagation using a lubrication-theory-based model.

Recently, Kolliopoulos et al.^15^ used a combination of theory and experiment to examine capillary-flow dynamics in open rectangular microchannels with negligible gravitational effects. Scanning electron microscopy and profilometry were used to highlight the complexity of the free-surface morphology. A self-similar lubrication-theory-based model accounting for this complexity was developed and model predictions were compared to those from the widely used MLW, as well as experimental observations over a wide range of channel aspect ratios *λ* and equilibrium contact angles *θ*_0_.

**Fig. 2:**
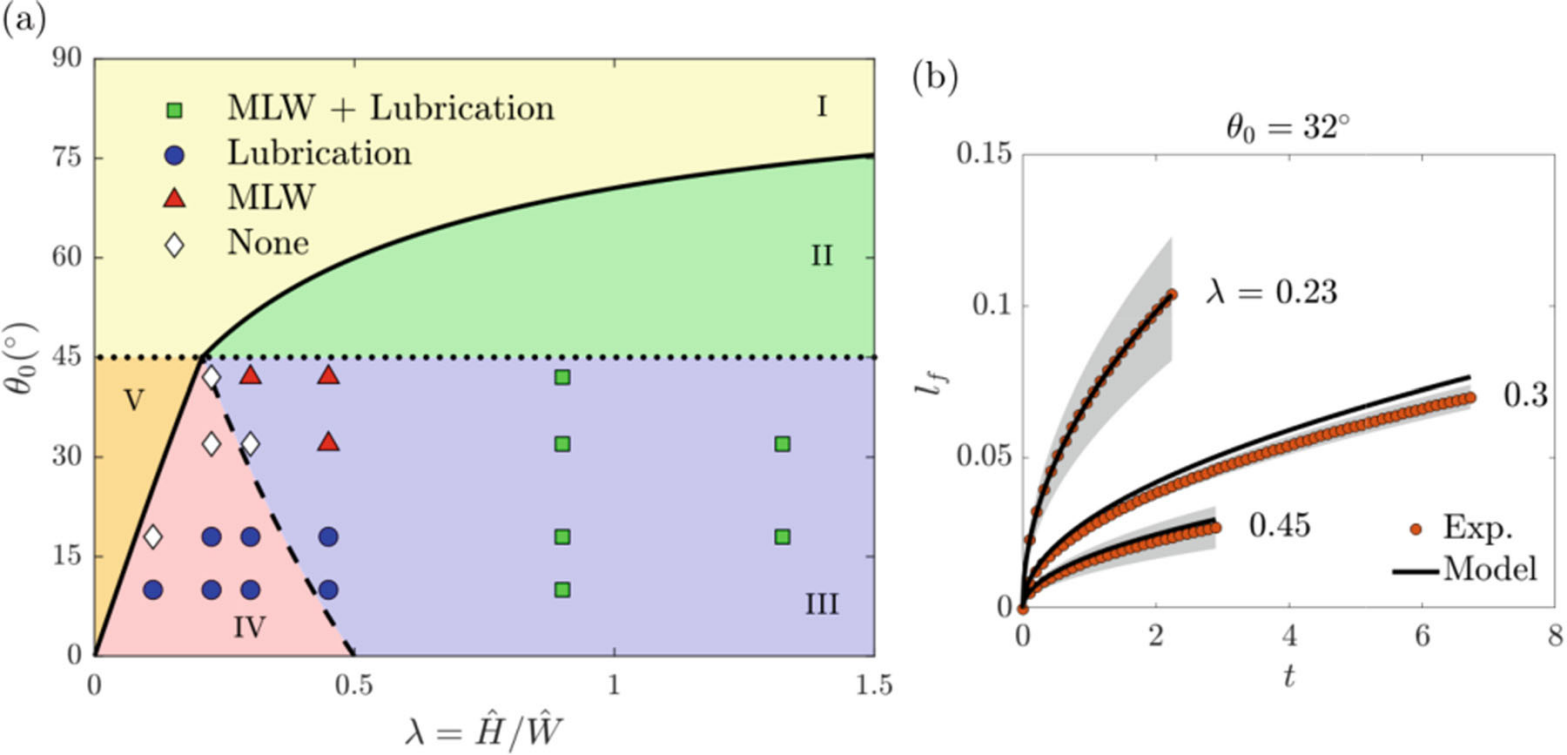
Free-surface morphology and finger length. **a** Free-surface morphology diagram as a function of equilibrium contact angle *θ*_0_ and channel aspect ratio *λ*. Solid symbols represent an agreement between lubrication-theory-based model and MLW model predictions with experiments^15^. The solid line corresponds to the limit where the capillary flow of the meniscus is energetically favorable in an open rectangular channel^54^. The dotted line corresponds to the contact-angle limit below which fingers form^47^. The dashed line corresponds to the critical aspect ratio at which fingers transition from a pinned to an unpinned state at the top of channel sidewall^15,54^. Region I has no capillary flow of the meniscus, region II has no fingers present, region III has fingers that are not pinned to the top of the channel sidewall, region IV has fingers that are pinned to the top of the channel sidewall, and region V has a capillary flow of fingers but not of the meniscus. **b** Effect of aspect ratio *λ* on dimensionless finger length ${l}_{\rm{f}}={\hat{l}}_{\rm{f}}/\hat{L}$ as a function of dimensionless time $t=\hat{t}\hat{H}\hat{\sigma }/{\hat{L}}^{2}\hat{\mu }$, where $\hat{L}$ is the channel length^15^. Solid symbols and shaded areas represent the average and range of experimental results, respectively. Solid lines represent lubrication-theory-based model predictions.

In Fig. 2a, model predictions are compared to experimental observations and the limitations of each model are identified based on the combination of channel aspect ratio *λ* and equilibrium contact angle *θ*_0_. Results are superimposed on the free-surface morphology regions identified by Seemann et al.^54^ using a combination of atomic force microscopy experiments and Surface Evolver modeling. For large *λ* the two model predictions are indistinguishable, whereas for smaller *λ* the lubrication-theory-based model agrees better with experiments. The lubrication-theory-based model is in better agreement with experiments at smaller *θ*_0_, although as *θ*_0_ → *π*/4 it fails to account for important axial curvature contributions to the free surface and the agreement worsens. The MLW model agrees with experiments for larger *λ* and larger *θ*_0_, where the contributions from the upper liquid–air interface are not as significant since the model assumes a flat upper liquid–air interface. However, for smaller *λ* the agreement worsens because the upper liquid–air interface contribution becomes significant.

The lubrication-theory-based model also quantitatively predicts the dynamics of fingers that extend ahead of the meniscus for a range of channel aspect ratios *λ* (Fig. 2b) and equilibrium contact angles *θ*_0_ (not shown here)^15^. These findings elucidate the limitations of the MLW model and demonstrate the importance of accounting for the effects of complex free-surface morphology on capillary-flow dynamics in open rectangular microchannels.

## Influence of evaporation

In addition to the complex free-surface morphology, the lack of a top allows evaporation to significantly affect flow if the liquid evaporates. In applications such as microfluidic devices used for diagnostic testing, evaporation can undesirably influence test results^5^. In contrast, in applications such as flexible printed electronics fabrication, evaporation is exploited to print conductive inks on flexible substrates that can be integrated with roll-to-roll manufacturing processes, potentially resulting in low-cost and high-throughput device fabrication. A relatively recent example of this is the self-aligned capillarity-assisted lithography for electronics (SCALE) process^1–3,55^.

The SCALE process initially involves using imprint lithography to mold a network of channels and reservoirs into a coated thermoset material (i.e., plastic substrate). An electronic ink is then sequentially deposited in the reservoirs via inkjet printing, and the ink flows into the channels due to capillary forces. During flow, the ink solvent evaporates, leading to the deposition of conductive material onto the channel sidewalls and bottom. This process is repeated using different liquids with successive drying steps to create a variety of electronic devices including resistors, capacitors, and transistors^1–3,55^. The quality and performance of the electronic devices are highly dependent on the simultaneous capillary flow and evaporation of the electronic inks. Hence, developing a fundamental understanding of flow and evaporation in open microchannels is vital to improving and optimizing such applications.

Motivated by thermal management devices such as heat pipes, previous studies have considered the effects of evaporation on steady flow in open rectangular^52,56^ and V-shaped^57–60^ channels. In these studies, the flow reaches a steady state when the capillary flow is balanced by evaporation, assuming an infinite supply of liquid at the channel inlet. The influence of evaporation on flow in V-shaped channels was recently examined theoretically by Gambaryan-Roisman^61^ using a diffusion-limited evaporation model. However, these previous studies focus on the flow of pure liquids, whereas most applications rely on the capillary flow of liquid solutions or colloidal suspensions.

Experiments on the capillary flow of nano- and micro-particle suspensions in open microchannel networks subject to evaporative lithography were conducted by Lone et al.^62^. After the flow reached the end of the channel, the carrier liquid was evaporated, resulting in deposition of the suspended particles on the channel bottom, creating a two-dimensional continuous metal pattern. Although particle suspensions were considered, the meniscus position in the experiments was found to scale as ${\hat{z}}_{\rm{M}} \sim {\hat{t}}^{1/2}$, consistent with what is expected for particle-free liquids in the absence of evaporation during flow. The influence of evaporation during flow (i.e., prior to the flow reaching the end of the channel) was not investigated in the experiments.

One of the first studies to examine the effects of evaporation on flow dynamics in open rectangular microchannels with negligible gravitational effects was conducted by Lade et al.^37^. Experiments were conducted using aqueous polymer solutions and electronic inks commonly found in printed electronics fabrication. The rate of evaporation was controlled using a humidity chamber and the meniscus position as a function of time was reported for a range of channel aspect ratios *λ* (height/width) and evaporation rates. Lade et al. also observed nonuniform solute deposition patterns after solvent evaporation, with solute accumulating near the front meniscus in a way that resembles the coffee-ring effect commonly observed in evaporating droplets. Strong disagreement was found between the experimental results and the predictions of a MLW model neglecting evaporation, which demonstrated the need for a model that accounts for the effects of evaporation on flow dynamics.

**Fig. 3:**
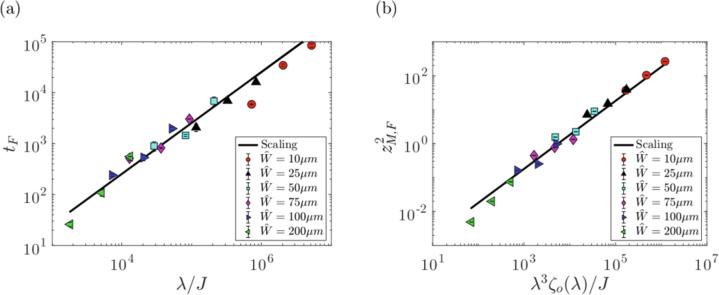
Scaling relationships. Scaling relationships for (**a**) dimensionless final time ${t}_{\rm{F}}={\hat{t}}_{\rm{F}}\hat{\sigma }/\hat{\mu }\hat{W}$ and (**b**) square of the dimensionless final meniscus position ${z}_{{\rm{M}},{\rm{F}}}^{2}={\hat{z}}_{{\rm{M}},{\rm{F}}}^{2}/{\hat{W}}^{2}$, for different channel widths $\hat{W}$^14^. The solid lines represent the proposed scaling relationships and the solid symbols represent experimental results by Lade et al.^37^. All channel heights $\hat{H}$ are 46.8 μm. The channel aspect-ratio function *ζ*_0_(*λ*) can be found in the work of Ouali et al.^24^.

A Lucas–Washburn-type one-dimensional model that incorporates the effects of a concentration-dependent viscosity and uniform evaporation on capillary flow in rectangular microchannels was developed by Kolliopoulos et al.^14^. The model yields predictions of the meniscus position evolution down the microchannel length. Model predictions were compared to the experiments by Lade et al.^37^ involving aqueous poly(vinyl alcohol) solutions by using the evaporation rate as a fitting parameter. The model qualitatively captures the meniscus position evolution for different relative humidities. However, the evaporation rates used to fit the model are *O*(10 − 10^2^) larger than those obtained from bulk drying experiments. This discrepancy was attributed to assuming a flat free surface and not accounting for axial concentration gradients in the model. Despite this discrepancy, scaling relationships obtained from the model for the dependence of the final meniscus position *z*_M,F_ and total flow time *t*_F_ on the channel dimensions and rate of uniform evaporation *J* are in good agreement with the experimental observations of Lade et al.^37^ (Fig. 3).

Solute concentration gradients arise during the flow of an evaporating liquid solution or colloidal suspension as demonstrated by Lade et al.^37^. These solute concentration gradients are expected to lead to surface-tension gradients, which in turn give rise to Marangoni stresses that act on the liquid to drive flow. Wijnhorst et al.^63^ used experiments to study the effect of surfactants on the dynamics of capillary rise and finger formation in closed rectangular channels and capillary tubes. The addition of surfactant results in reduced meniscus propagation due to the decrease in surface tension, and qualitative differences were observed in finger dynamics compared to pure liquids. The results suggest that surfactants can either enhance and inhibit the finger dynamics depending on the choice of surfactant. While these experiments were conducted using closed channels, where the flow is driven by the pressure gradient caused by the circular-arc meniscus front, for open channels the curvature gradients due to the additional free surface also drive flow. Hence, we expect the addition of surfactants to have a greater influence on flow dynamics in open channels.

## Conclusions

Exploiting spontaneous capillary flow for the control and transport of liquids is of great importance for numerous in-space and terrestrial applications. While the capillary flow of pure nonvolatile liquids has been extensively investigated, liquid solutions or colloidal suspensions with volatile components or surfactants have received less attention despite many applications relying on such liquids. New insights into the effects of evaporation and surfactants on the capillary flow of multicomponent liquids have practical implications for numerous applications, such as lab-on-a-chip devices, heat pipes, propellant management devices in spacecrafts, paper-based microfluidics, fuel cells, porous-media flows, and fabrication of flexible printed electronics. Comprehensive physical understanding of this topic will likely involve a close interplay between experiments, analytical and semi-analytical models, and direct numerical simulations.

## Data Availability

All data analyzed during this study are included in this published article.

## Code availability

No custom computer codes or algorithms were used to generate new results for this paper.
